# Polymicrobial periodontal disease triggers a wide radius of effect and unique virome

**DOI:** 10.1038/s41522-020-0120-7

**Published:** 2020-03-10

**Authors:** Li Gao, Misun Kang, Martin Jinye Zhang, M. Reza Sailani, Ryutaro Kuraji, April Martinez, Changchang Ye, Pachiyappan Kamarajan, Charles Le, Ling Zhan, Hélène Rangé, Sunita P. Ho, Yvonne L. Kapila

**Affiliations:** 1 Department of Orofacial Sciences, School of Dentistry, University of California San Francisco, San Francisco, CA USA; 2 Department of Periodontology, Guanghua School of Stomatology, Hospital of Stomatology, Sun Yat-Sen University, Guangzhou, China; 3 Department of Preventive and Restorative Dental Sciences, School of Dentistry, University of California San Francisco, San Francisco, CA USA; 4 Oralome, Inc., QB3 labs, UCSF Mission Bay Campus, Byers Hall, San Francisco, CA USA; 5 Department of Life Science Dentistry, The Nippon Dental University, Tokyo, Japan; 6 Department of Periodontology, The Nippon Dental University School of Life Dentistry at Tokyo, Tokyo, Japan; 7 State Key Laboratory of Oral Diseases, National Clinical Research Center for Oral Diseases, Department of Periodontology, West China Hospital of Stomatology, Sichuan University, Chengdu, China; 8 Department of Periodontology, Université de Paris, Faculty of Odontology; APHP, Rothschild Hospital, Paris, France; 9 EA2496, Université de Paris, Faculty of Dental Surgery, Montrouge, France; 10 Department of Urology, School of Medicine, University of California San Francisco, San Francisco, CA USA

**Keywords:** Pathogens, Plaque

## Abstract

Periodontal disease is a microbially-mediated inflammatory disease of tooth-supporting tissues that leads to bone and tissue loss around teeth. Although bacterially-mediated mechanisms of alveolar bone destruction have been widely studied, the effects of a polymicrobial infection on the periodontal ligament and microbiome/virome have not been well explored. Therefore, the current investigation introduced a new mouse model of periodontal disease to examine the effects of a polymicrobial infection on periodontal ligament (PDL) properties, changes in bone loss, the host immune response, and the microbiome/virome using shotgun sequencing. Periodontal pathogens, namely *Porphyromonas gingivalis, Treponema denticola, Tannerella forsythia*, and *Fusobacterium nucleatum* were used as the polymicrobial oral inoculum in BALB/cByJ mice. The polymicrobial infection triggered significant alveolar bone loss, a heightened antibody response, an elevated cytokine immune response, a significant shift in viral diversity and virome composition, and a widening of the PDL space; the latter two findings have not been previously reported in periodontal disease models. Changes in the PDL space were present at sites far away from the site of insult, indicating that the polymicrobial radius of effect extends beyond the bone loss areas and site of initial infection and wider than previously appreciated. Associations were found between bone loss, specific viral and bacterial species, immune genes, and PDL space changes. These findings may have significant implications for the pathogenesis of periodontal disease and biomechanical properties of the periodontium. This new polymicrobial mouse model of periodontal disease in a common mouse strain is useful for evaluating the features of periodontal disease.

## Introduction

Periodontitis is an inflammatory disease triggered by a microbial dysbiosis that affects the supporting tissues around teeth and it eventually leads to tooth loss if left untreated. Periodontitis has been associated with an increased risk of diabetes mellitus, cardiovascular diseases, adverse pregnancy outcomes, respiratory infections, and rheumatoid arthritis^1,2^. A dysbiotic oral microbiota is the initiating factor in the pathogenesis of periodontitis that leads to a dysregulated host immune response^3,4^.

There are more than 700 species colonizing the oral cavity^5^ and hundreds of those can be present within oral biofilms^6,7^. Among the biofilm-associated microbiota, several bacterial species are highly associated with periodontal disease; namely *Porphyromonas gingivalis*, *Treponema denticola*, and *Tannerella forsythia*. This pathogenic consortium of microbes is known as the “Red Complex”^8^ due to their strong association with clinical parameters of severe periodontal disease, such as periodontal pocket depths and bleeding on probing^8–11^. However, recent studies using high throughput sequencing approaches are shedding new light on novel bacterial signatures and a larger and more diverse microbiome associated with periodontal disease^12,13^. Another gram-negative bacterium, namely *Fusobacterium nucleatum*, which belongs to the “orange complex” and is closely related to the red complex, is also associated with periodontitis^8^. *F. nucleatum* can coaggregate with a significant number of bacteria in the oral cavity and is thought to be an important “microbial bridge” during dental plaque formation^14,15^. These pathogenic bacteria contribute to periodontal disease via a variety of mechanisms, including through secretion of proteolytic enzymes or proteases^16^, activation and modulation of the host immune response via bacterial LPS and other surface effector molecules^17,18^, and invasion of host cells^19^. These molecular and cellular processes lead to destruction of the periodontal supporting tissues.

Although viruses are also present within the oral cavity with differences observed between health and disease states, there is limited knowledge about their relationship to periodontal disease^20^. Studies suggest that viruses, including herpes simplex virus-1,2, Epstein–Barr virus, and cytomegalovirus play important roles in periodontal disease^21–29^. Also, in recent studies, other different viruses have been associated with periodontal disease, however, a comprehensive evaluation of the oral virome in relationship to periodontal disease using shotgun sequencing has not been carried out in mice and to a limited extent in humans^30–33^.

The effects of a defined polymicrobial infection on periodontal ligament properties and on the microbiome/virome have not been examined. Thus, in this study, a new polymicrobial mouse model of periodontal disease, induced by oral infection with *P. gingivalis*, *T. denticola*, *T. forsythia*, and *F. nucleatum* in a common mouse strain, was introduced to examine the effects on periodontal ligament properties and the microbiome/virome.

## Results

### A polymicrobial oral infection can be successfully obtained in a common mouse strain

**Fig. 1:**
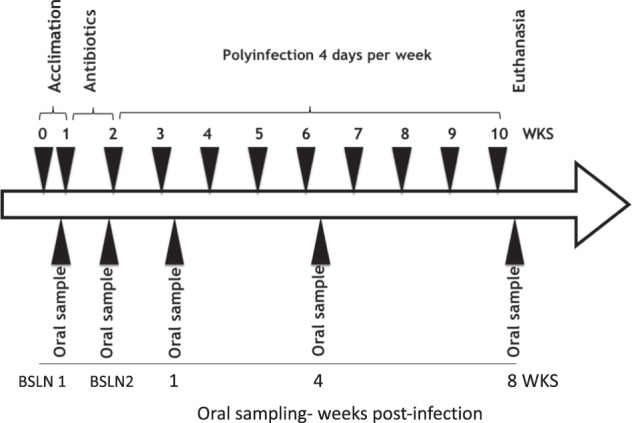
Mouse infection and oral sample collection timeline. Eight polymicrobial infection sessions were carried out once per week from the 3rd to the 10th week. Oral swab samples were collected on the day before antibiotic treatment, the day before infection and at 1, 4, and 8 weeks following the initial infection. Blood and tissue specimen collection was performed at euthanasia following 8 weeks of infection.

**Fig. 2:**
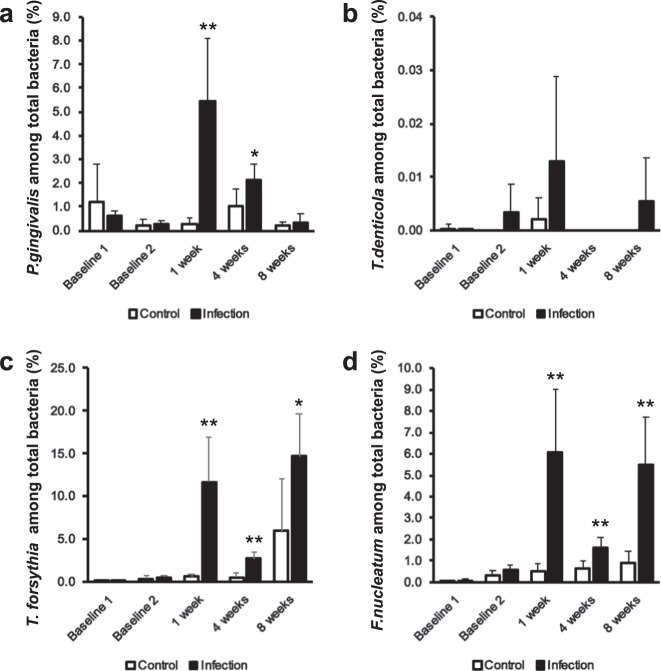
A polymicrobial oral infection can be successfully obtained in a common mouse strain. Oral swab samples were collected from the teeth, oral mucosa, and gingiva of mice with micro cotton tip swabs to evaluate microbial status of the polymicrobial infection model. DNA was isolated and purified from the swab samples, which were collected at different time points (baseline 1; before infection and before an antibiotic wash out; baseline 2; before infection and after an antibiotic washout, and 1, 4, and 8 weeks after infection) and then the four periodontal pathogens (*P. gingivalis*, *T. denticola*, *T. forsythia*, and *F. nucleatum*) and total bacteria were quantified by standard real-time PCR using primers corresponding to 16S ribosomal RNA. **a**–**d** The data are shown as a percentage of each pathogen among total bacteria; mean ± s.d.

A polymicrobial infection model of periodontal disease in a commonly employed mouse strain (BALB/cByJ) was used to evaluate periodontal bone loss and related parameters (Fig. 1). A polymerase chain reaction (PCR)-based approach was used to confirm oral infection with all four periodontal pathogens *(P. gingivalis*, *T. denticola*, *T. forsythia*, and *F. nucleatum*) in this strain of mice (Fig. 2; Table 1). The oral swab results indicated that all four bacteria were detectable throughout the experimental period from 1 to 8 weeks post infection; except *T. denticola*, which was always present except at 4 weeks. All pathogens were present at higher levels in the infected versus control group for all time points. These differences were statistically significant for all pathogens except *T. denticola*, which showed a trend toward higher levels compared to controls. The highest levels of the four pathogens were detected at 1 week post infection, except for *T. forsythia*, which showed even higher levels at 8 weeks. *T. denticola* was not detected at 4 weeks in either infection or control group and at 8 weeks it was only detected in the infection group. The number of mice exhibiting infection with the different pathogens varied. For example, *P. gingivalis* was present in all mice at all time points. However, *T. denticola* was present in generally few mice throughout the study period. *T forsythia* and *F. nucleatum* were similar to *P. gingivalis* in that they were present in most mice throughout the study.

**Table 1:** Bacterial content from oral swabs.

	*P. gingivalis*		*T. denticola*		*T. forsythia*		*F. nucleatum*		Total bacteria	
	Control	Infection	Control	Infection	Control	Infection	Control	Infection	Control	Infection
Baseline 1	16,981.09 ± 28,369.48	723.03 ± 407.71	1.18 ± 2.05	0.07 ± 0.07	8.43 ± 14.57	31.56 ± 23.59	59.47 ± 11.56	72.66 ± 28.24	553,248.72 ± 639,763.52	121,168.24 ± 80,829.30
	(4:4; 100%)	(6:6; 100%)	(1:4; 25%)	(2:6; 33%)	(2:4; 50%)	(5:6; 83%)	(4:4; 100%)	(6:6; 100%)	(−)	(−)
Baseline 2	58.39 ± 85.52	77.71 ± 61.10	0.00 ± 0.00	1.03 ± 1.46	103.48 ± 54.06	151.63 ± 42.29	104.50 ± 36.60	149.37 ± 48.89	37,282.08 ± 16,477.93	30,052.84 ± 7,525.85
	(6:6; 100%)	(6:6; 100%)	(0:6; 0%)	(2:6; 33%)	(6:6; 100%)	(6:6; 100%)	(6:6; 100%)	(6:6; 100%)	(−)	(−)
1 week	299.89 ± 100.69	4,077.78 ± 1,805.15	1.19 ± 1.87	7.26 ± 4.58	1,096.65 ± 832.67	8,935.11 ± 3,838.25	550.51 ± 232.78	4,547.68 ± 2,009.55	183,983.00 ± 115,301.42	92,192.86 ± 50,283.58
	(5:5; 100%)	(5:5; 100%)	(2:5; 40%)	(4:5; 80%)	(5:5; 100%)	(5:5; 100%)	(5:5; 100%)	(5:5; 100%)	(−)	(−)
4 weeks	191.06 ± 68.67	15,943.21 ± 13,755.35	0.00 ± 0.00	0.00 ± 0.00	88.87 ± 75.33	21,464.59 ± 18,251.40	120.49 ± 29.79	12,066.57 ± 10,766.24	21,150.11 ± 5,938.39	746,180.73 ± 576,242.33
	(6:6; 100%)	(6:6; 100%)	(0:6; 0%)	(0:6; 0%)	(6:6; 100%)	(6:6; 100%)	(6:6; 100%)	(6:6; 100%)	(−)	(−)
8 weeks	73.40 ± 66.95	728.94 ± 1,059.93	0.00 ± 0.00	10.62 ± 15.06	2,005.86 ± 2,431.41	27,481.09 ± 5,043.55	296.09 ± 212.52	10,280.58 ± 2,788.89	32,852.47 ± 12,385.56	204,945.44 ± 67,419.62
	(6:6; 100%)	(6:6; 100%)	(0:6; 0%)	(2:6; 33%)	(6:6; 100%)	(6:6; 100%)	(6:6; 100%)	(6:6; 100%)	(−)	(−)

Actual numbers of bacteria/swab (mean ± s.d.)

(presence of periodontal pathogens in numbers of mice [%]).

### The polymicrobial infection induces a host antibody response against all periodontal pathogens

**Fig. 3:**
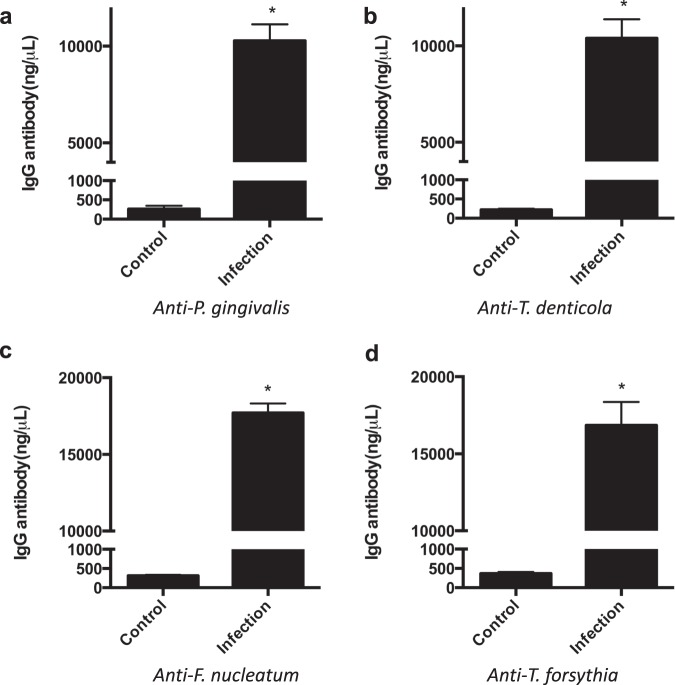
Serum IgG antibody response to *P. gingivalis*, *T. denticola*, *T. forsythia*, and *F. nucleatum* inoculation in Control and Infection groups. Data represent the means ± s.d. from six mice per group. *, the difference in serum IgG antibody levels for (**a**) *P. gingivalis*, (**b**) *T. denticola*, (**c**) *F. nucleatum*, and (**d**) *T. forsythia* was significant (*p* < 0.001) compared to the Control group.

To further validate the infection and to evaluate the systemic host response to the polymicrobial infection, serum antibody levels to the four periodontal pathogens were evaluated using an ELISA approach (Fig. 3). Infected mice showed a significant IgG antibody response to all four periodontal pathogens compared to the uninfected control mice. Specifically, the IgG antibody levels for *P. gingivalis* were 10,279.13 ng/ml ± 845.68 in the infected group compared to 257.37 ng/ml ± 87.18 in the control. The IgG antibody levels for *T. denticola* were 10,395.19 ng/ml ± 975.65 in the infected group compared to 218.21 ng/ml ± 25.76 in the control. The IgG antibody levels for *F. nucleatum* were 17,702.02 ng/ml ± 614.57 in the infected group compared to 310.64 ng/ml ± 27.19 in the control. The IgG antibody levels for *T. forsythia* were 16,848.83 ng/ml ± 1509.53 in the infected group compared to 367.21 ng/ml ± 41.82 in the control.

### The polymicrobial infection induces a significant change in immune and cytokine gene expression

To further characterize this polymicrobial mouse model, immune gene profiling was performed on the gingival tissues of the infected and control mice. Using an RNA array of 84 genes, this profiling revealed 18 genes that were differentially altered 1.5-fold or greater in the infected mice compared to the control uninfected mice (Table 2), although *p* values were greater than 0.05. The most differentially elevated genes, included secretoglobin gene super family cytokine (8.473-fold change) and interferon gamma-inducing factor (2.287-fold change), and the most differentially decreased gene was tumor necrosis factor ligand 1C (−2.336-fold change). The *Scgb3a1* gene encodes a small secreted protein that may function in the inhibition of cell growth. Expression of the related gene in humans is downregulated via DNA methylation in breast cancers and other tumor types. Alternatively, spliced transcript variants encoding multiple isoforms have been observed for this gene^34^.

**Table 2:** Immune gene profiling of oral tissues following polymicrobial infection in mice.

Gene	RefSeq	Description	Fold Change	*p* Value
Scgb3a1	NM_170727	Secretoglobin gene super family cytokine; HIN-1	8.473	0.191
Il18	NM_008360	Interferon gamma-inducing factor	2.287	0.069
Csf3	NM_009971	Granulocyte colony-stimulating factor	1.782	0.126
Il9	NM_008373	T-cell growth factor P40; cytokine P40	1.732	0.187
Il17b	NM_019508	Neuronal interleukin-17-releated factor	1.647	0.264
Il13	NM_008355	T-cell activation protein P600	1.643	0.297
Il3	NM_010556	Multipotential colony-stimulating factor	1.623	0.359
Il20	NM_021380	Four helix bundle cytokine 10; IL-20	1.601	0.380
Ifna2	NM_010503	Interferon alpha family, gene 2	1.592	0.345
Il6	NM_001314054	B-cell hybridoma growth factor; IL HP-1	1.583	0.270
Gdf15	NM_011819	Macrophage inhibiting cytokine-1	1.581	0.189
Il10	NM_010548	Cytokine synthesis inhibitory factor	1.568	0.312
Il21	NM_021782	Interleukin-21 isoform 2 precursor	1.559	0.267
Spp1	NM_009263	Early T-lymphocyte activation 1 protein	1.549	0.309
Ifna4	NM_010504	Interferon alpha family, gene 4	1.528	0.408
Bmp5	NM_007555	Bone morphogenetic protein 5 preproprotein	1.523	0.064
Osm	NM_001013365	Oncostatin-M precursor	1.517	0.314
Ltb	NM_008518	Tumor necrosis factor ligand 1C	−2.336	0.106

### Alveolar bone loss and intrabony defects are significantly increased with a polymicrobial infection

**Fig. 4:**
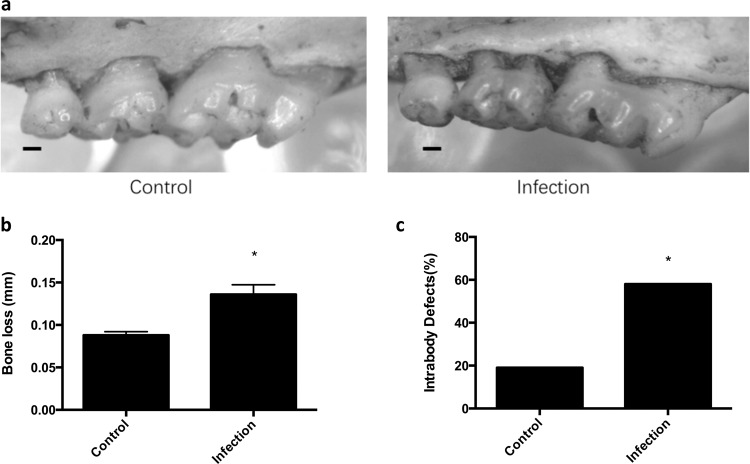
Alveolar bone loss is significantly increased with the polymicrobial infection. **a** Representative images of alveolar bone loss on the palatal surfaces of maxillary molars in control and infection groups. Scale bar represents 0.2 mm. **b** The graphs represent alveolar bone loss in control mice or mice with the polymicrobial infection (Infection). Data represent the means ± s.d. from six mice per group. For each mouse, alveolar bone loss was calculated as the average from 28 sites (3 sites on the first molar, 2 sites on the second molar, and 2 sites on the third molar, on both sides of the left maxilla and mandible). *, the difference in alveolar bone loss was significant (*p* < 0.001) compared to the Control group. **c** The percentage of intrabony defects was calculated as the number of tooth surfaces containing periodontal intrabony defects out of total tooth surfaces. For each group, there were a total of 72 tooth surfaces (6 mice, 36 molars, 72 sides (buccal, palatal/lingual)). *, the difference in the percentage of intrabony defect was significant (*p* < 0.001) compared to the Control group.

Once the local infection with and systemic response to the four periodontal pathogens was confirmed, the alveolar bone response to the polymicrobial infection was evaluated in the mice. Bone loss was evaluated in the maxillary and mandibular molars of the mice. After 8 weeks of inoculation and infection with the periodontal pathogens, mice exhibited significantly more alveolar bone loss (0.136 mm ± 0.011) compared to the control group (0.088 mm ± 0.004) (Fig. 4a, b).

The presence of alveolar intrabony defects was also evaluated following infection. Infected mice showed significantly more intrabony defects (42/72 or 58% of sites) compared to control uninfected mice (14/72 or 19% of sites) (Fig. 4c).

### The polymicrobial infection induces a widening of the periodontal ligament space throughout, thereby inducing a broad radius of effect

**Fig. 5:**
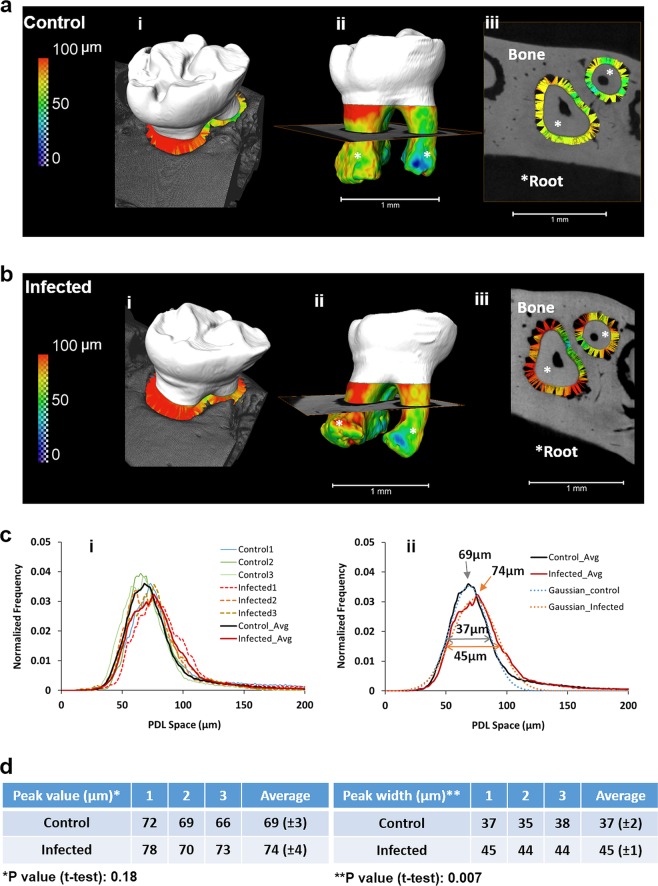
The periodontal ligament (PDL) space in infected mice was significantly wider when compared to the PDL space in controls. **a**, **b** The PDL-space was measured as the distance between the tooth and bone in control (**a**i) and infected (**b**i) groups. PDL space is shown as a color map on the root surface of the control (**a**ii) and infected (**b**ii) teeth, and as a pseudo ligament in virtual sections of both control (**a**iii), and infected (**b**iii) teeth. Color maps are indicative of distance between roots (*) of molars and alveolar bone of the periodontal complexes of control (**a**) and infected (**b**) mice. **c** Distribution of PDL spaces in control (solid) and infected (dotted) mice (i) indicate that PDL-spaces in infected mice have a larger variance (broaden peak) and a widened PDL space compared to lower variance (narrower peak) and uniform PDL space in controls (ii). **d** Left and right tables demonstrate peak values and peak widths of the PDL space in control (top row) and infected (bottom row) mice. No significant difference in peak values of respective PDL spaces between groups was observed (*p* = 0.18). However, a significant difference between peak widths (*p* = 0.007) indicated that the PDL-space in infected mice on average was significantly widened (>100 µm) compared to that of controls. Data represent mean ± s.d.

Given that the role of periodontal pathogens in modulating periodontal ligament parameters has not been previously examined, this study evaluated the dimensions of the periodontal ligament space following infection with the four periodontal pathogens. The polymicrobial infection triggered a widening of the periodontal ligament space throughout its length as measured by micro-CT analysis (Fig. 5). Specifically, the infected group exhibited significantly increased periodontal ligament space/width (45 μm ± 1) compared to the control group (37 μm ± 2). Notably, the widened periodontal ligament space was detected throughout the periodontal ligament and even at a great distance from the site of infection (gingival margin) and bone loss thereby inducing a broad radius of effect.

### The polymicrobial infection induces significant changes in viral diversity and the virome

**Fig. 6:**
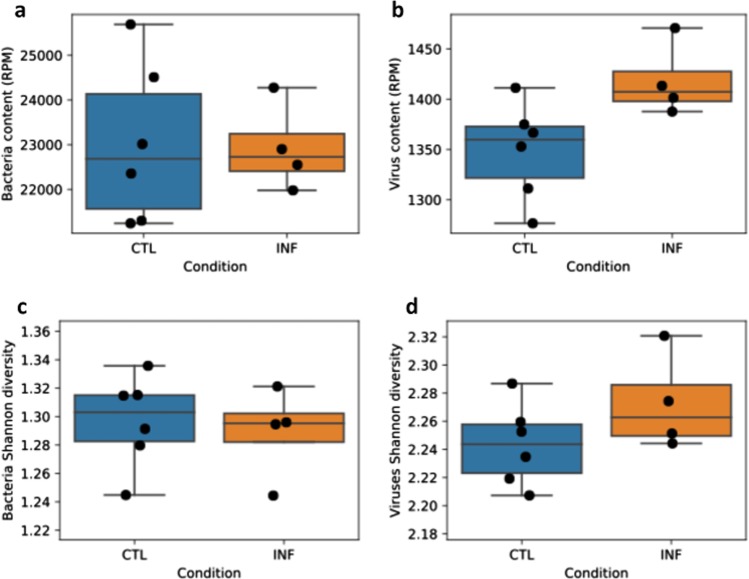
Polymicrobial periodontal disease is associated with increased viral diversity. These panels demonstrate the comparison of infected and control mice based on bacterial and viral content. Panel **a** shows the read content (reads per million) based on bacterial species and panel **b** shows the read content (reads per million) based on viral species. Panels **c** and **d** show the bacterial and viral diversity, respectively. In panel **b**, the difference in the infected (INF) group was significant (*p* < 0.05) compared to the control (CTL) group. Data is represented using boxplots.

For the first time, a metagenomic shotgun sequencing approach was used to examine the microbiome and virome of periodontal disease in a mouse model with a defined polymicrobial infection. Analysis of the gingival microbiome/virome data revealed that there is a significant increase in viral content and diversity in response to the polymicrobial infection in mice (Fig. 6). Although, the bacterial content and diversity was not significantly different. Specifically, the polymicrobial infection group had similar bacterial reads (panel a, *p* value 0.92) but significantly more virus reads (panel b, *p* value = 0.04) compared to the control group. In addition, the infection group had a slight decrease in bacterial diversity (panel c, *p* value = 0.72) and a slight increase in viral diversity (panel d, *p* value = 0.18) compared to the control group.

### Specific viral and bacterial species are associated with bone loss and changes in PDL space

**Fig. 7:**
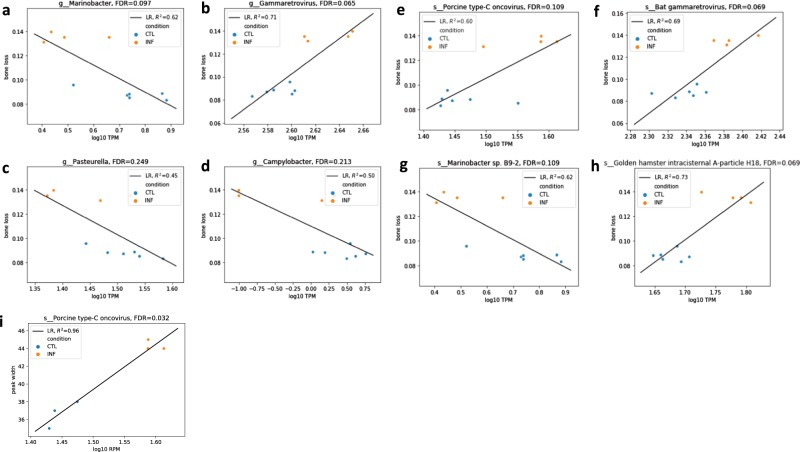
Specific microbial and viral species are associated with bone loss and increased PDL space. The graphs show the top microbial and viral species that correlate with the level of bone loss (**a**–**h**). The *x*-axes show the abundance of microbes/viruses and the *y*-axes shows the level of bone loss in mm. The graph in panel **i** shows the virus that is most highly associated with and an increased PDL space.

Specific viral and bacterial species were associated with bone loss in mice with the polymicrobial infection (Fig. 7a–h; Table 3). Specifically, genus Marinobacter (false discovery rate (FDR) = 0.097, *R*^2^ = 0.62, decreases with bone loss), genus Gammaretrovirus (FDR = 0.065, *R*^2^ = 0.71, increases with bone loss), genus Pasteurella (FDR = 0.249, *R*^2^ = 0.45, decreases with bone loss), genus Camphylobacter (FDR = 0.213, *R*^2^ = 0.50, decreases with bone loss), species Porcine type-C oncovirus (FDR = 0.109, *R*^2^ = 0.60, increases with bone loss), species Bat gammaretrovirus (FDR = 0.069, *R*^2^ = 0.69, increases with bone loss), species Marinobacter sp B9-2 (FDR = 0.109, *R*^2^ = 0.62, decreases with bone loss), and Golden hamster intracisternal A particle H18 (FDR = 0.069, *R*^2^ = 0.73, increases with bone loss) were associated with bone loss.

**Table 3:** Bacteria and viruses significantly associated with bone loss and periodontal ligament width changes.

	Slope (bone loss ~log10 TPM)	FDR
*Bacteria/viruses significantly increase with bone loss*		
g__Gammaretrovirus	0.768797	0.065039
s__Golden hamster intracisternal A-particle H18	0.362623	0.0692544
s__Bat gammaretrovirus	0.640580	0.0692544
s__Porcine type-C oncovirus	0.261559	0.108691
*Bacteria/viruses significantly decrease with bone loss*		
g__Campylobacter	−0.027827	0.212951
g__Pasteurella	−0.241754	0.249187
g__Marinobacter	−0.112225	0.0970535
s__Marinobacter sp. B9-2	−0.112225	0.108691

	Slope (peak width ~log10 TPM)	FDR
*Bacteria/viruses significantly increase with peak width*		
s__Porcine type-C oncovirus	50.851206	0.031868

The species Porcine type-C oncovirus (FDR = 0.032, *R*^2^ = 0.96, increases with peak width) was associated with increased PDL space or (peak width) (Fig. 7i).

In addition, we studied the relationship between the microbial environment and the immune response and found some weaker associations between bacteria/virus species and immune genes (Supplemental Fig. 1). The species Gibbon ape leukemia virus (FDR = 0.2602, *R*^2^ = 0.78, increases with gene-expression level) was associated with the immune gene Tnfsf14 and the species Clostridiales bacterium 1 7 47FAA) was associated with the immune gene Adipoq (FDR = 0.2602, *R*^2^ = 0.88, increases with gene-expression level). Also, there were weaker associations between bacteria/virus species and PDL space (Supplemental Fig. 2).

## Discussion

Several different mouse models of periodontal disease that mimic the human condition have been proposed in the literature, including ligature models^35,36^, injection models^37,38^, mono-infection models inoculated with only one strain of bacteria^39–42^, and even polymicrobial infection models^43–45^. Ligature and injection models develop aggressive lesions, and the destruction of periodontal tissues is always localized and rapid. These characteristics do not mimic the chronic nature of periodontal disease. Furthermore, mono-infection models do not recapitulate the polymicrobial nature of periodontal disease. Several studies have suggested that the host immune and physiologic responses are synergistic and interactive in response to a polymicrobial infection, which is distinct from responses to a mono-infection^46,47^. A polymicrobial infection model more closely approximates the natural pathogenesis of the disease. However, prior polymicrobial mouse models of periodontal disease used genetically and immune compromised/modified mouse strains. In an effort to develop a mouse model that more closely resembles the pathogenesis of periodontal disease, this study employed a common mouse strain (BALB/cByJ) with an intact immune system and not genetically altered, to induce periodontal disease with four key periodontal pathogens. Similar to other mouse models of periodontal disease^43,45,48^, this current model exhibits the classic signs of alveolar bone loss. The current model further shows the presence of intrabony defects.

Mouse models of periodontal disease have shown various levels of bacterial colonization efficiency similar to the present study^43,45,48,49^. The current study revealed that all four bacterial pathogens (*P. gingivalis*, *T. denticola*, *T. forsythia*, *F. nucleatum*) colonize the oral cavity of the infected mice based on the PCR data, although *T. denticola* is less effective. Also, given that the four pathogens are detected at different levels in the oral cavity and in different numbers of mice, this indicates a variable colonization efficiency among the different bacteria. For example, oral swab data (Fig. 2) show that *P. gingivalis*, *T. forsythia*, and *F. nucleatum* were detected throughout the experimental period, whereas *T. denticola* was not detected at 4 weeks, and when detected, it was not always present in all mice. *T. denticola* was present in low levels throughout the study; consistent with findings in human periodontal disease^50^. Parenthetically, our results (Fig. 2) show that the four human periodontal pathogens were detected in oral swabs at baseline in uninfected mice (Baseline 1). There may be a few reasons for this finding. Other studies have not detected human oral periodontal pathogens in uninfected control mice^43,45,48,49,51^ although these other studies used regular PCR methods, whereas the current study used the more sensitive quantitative real-time PCR (qRT-PCR) method of detection. Thus, we may have detected a false positive. Consistent with this observation is that the serum antibody response to the periodontal pathogens in the control uninfected groups was near zero, similar to other studies^48,52^. Another potential explanation is that these were not germ free mice and they may reveal a low level of contamination. For this reason, all mice were treated with antibiotics to eliminate bacterial load and carriage of contaminating species prior to the start of the study.

Demonstration of a serum antibody response to the bacterial inoculum supports the utility of a given mouse model of periodontal disease. Other mouse models have shown that mice mount an antibody response to *P. gingivalis*, *T. denticola*, *T. forsythia*, and *F. nucleatum*^43,45,48^. Similarly, our data confirmed that this mouse strain can be used to effectively mount an antibody response to all four periodontal pathogens when induced by oral inoculation. All four periodontal pathogens induce a systemic antibody response significantly higher than control mice. Although, serum antibody levels to the four periodontal pathogens were all significantly increased, serum levels of IgG levels against *T. forsythia*, and *F. nucleatum* were even higher than to *P. gingivalis* and *T. denticola*. In previous studies in genetically altered mice^43,45,48^, the serum IgG levels to these four periodontal pathogens were also differentially elevated. The bacterial colonization efficiency of the four pathogens (i.e., Fig. 2) and the altered immune response of the different mice strains may contribute to differences in serum antibody responses. In addition, the high levels of antibodies to *T. denticola* may suggest that *T. denticola* exhibits a high virulence level, which could induce a high immune response even in the presence of low colonization. Another possibility is that our sampling times may have missed high fluctuations in colonization by *T. denticola* following infection. This fluctuation in *T. denticola* levels was also noted in other studies^43,51^. Also, since the serum antibody response was cumulative (measured at the end of the experimental period) and the colonization detection was performed at different time points during the study, the antibody response to *T. denticola* reflects a high summative response at the end even though organism colonization fluctuated.

Other mouse models of periodontal disease have also shown an altered host immune response to a polymicrobial infection^43,45,48^. However, these mouse models utilized genetically altered (ApoE or integrin beta 6 null mice) mice and they highlighted a variety of altered immune and cytokine profiles in serum and in local or organ-specific tissues. Although, the current study also highlighted an elevated host immune cytokine response featuring 18 genes that were differentially altered (Table 2), these were generally different from those highlighted in other studies. Unique to this study is that the most differentially elevated genes, included secretoglobin gene super family cytokine (8.473-fold change) and interferon gamma-inducing factor (2.287-fold change), and the most differentially decreased gene was tumor necrosis factor ligand 1C (−2.336-fold change). Although, interferon gamma and tumor necrosis factor family members have been implicated in such models, the secretoglobin gene super family cytokine has not been highlighted previously in any models of periodontal disease or inflammation; thus, warranting further study.

A long-held tenet in the relationship between periodontal disease pathogenesis and bacterial plaque has been the concept of a close or proximal radius of effect or radius of action^53–55^. This concept maintains that bacterial plaque can induce bone loss or mediate destructive effects on the periodontium only within a radius of 1.5–2.5 mm, but beyond 2.5 mm there is no effect. The current study, which shows a widening of the periodontal ligament space throughout the ligament and at a distance far removed from the site of bone loss and initial site of bacterial infection, supports a new concept—that the bacterially mediated radius of effect on the periodontium is far greater than previously thought, and it effects or alters deeper aspects of the periodontal ligament space. This latter finding may have significant implications for the pathogenesis of periodontal disease and biomechanical properties of the periodontium.

The virome, preferentially characterized using shotgun metagenomic sequencing, includes bacteriophages and viruses; both endogenous retroviruses and eukaryotic. The virome has recently been recognized as an essential part of the human microbiome, containing the most numerous and diverse microbial entities, and playing important roles in nutrient and energy cycling. Interactions between viruses, bacteria, and host cells have been associated with shifts from health to disease. The human virome has been implicated in the pathogenesis of several chronic diseases, including diabetes, inflammatory bowel disease, human immunodeficiency virus infection, and cancer^56^. The current study demonstrates a significant change in viral diversity and specific viruses associated with bone loss in the context of periodontal disease. In our study, periodontal disease is associated with an increase in the content and richness of mostly the retroviruses (Golden hamster intracisternal A-particle H18; Jaagsiekte sheep retrovirus; Mouse Intracisternal A-particle; Porcine type-C oncovirus; Murine leukemia-related retroviruses; Squirrel monkey retrovirus; Murine leukemia virus). Interestingly, endogenous retroviral proteins have been implicated in promoting autoimmune disease, neurodegenerative disorders, chronic inflammation, and cancer in humans. Indeed, human endogenous retroviruses express proteins that can elicit a dysregulated immune response. Eukaryotic viruses, such as herpes virus, may also stimulate endogenous retroviruses. Similar to our findings, an altered gut virome has been associated with the loss of gut mucosal integrity and activation of an innate immune response in the mouse intestine^57^. A previous study of saliva and pooled dental plaque from a limited number of patients (*n* = 7) with periodontal disease showed differences in virome composition between healthy/gingivitis/mild periodontitis patients and those with moderate/severe periodontal disease^20^. Another virome study in humans with limited periodontal samples identified a new family of redondoviridae associated with periodontal disease^31^. Taken in aggregate, the current study in mice with a well-defined polymicrobial infection/periodontal disease and the human studies suggest a potential role for an altered virome in the pathogenesis of periodontal disease.

The current data (Fig. 6) show that the polymicrobial infection induces significant changes in viral diversity and total virome load. In comparison, this data shows no significant difference in total bacterial load or in Shannon diversity score between the two conditions. However, this does not mean that the bacterial load and microbial diversity are the same between the two conditions. Specifically, although the shotgun sequencing data for the gingiva did not reveal significant differences in the total bacterial load or bacterial diversity, significant differences in the four periodontal pathogens as assessed by qRT-PCR in oral swabs (Fig. 2) and differences in the shotgun sequencing data of the gingiva for specific bacterial species (Fig. 7) indicate that bacterial shifts were indeed present within the oral microbiome of these mice. These shifts may not be detectable when assessing total bacterial load and bacterial diversity by shotgun sequencing. A larger sample size may have been helpful in this regard. Also, the microbiome of gingival tissue may not correlate precisely with that of the oral swabs. Studies have discussed issues with oral microbial sampling and detection methods in mouse models since oral murine tissues may exhibit lower bacterial biomass^58^. Mouse studies that have evaluated the oral microbiome with conventional 16S sequencing methods have detected some differences in oral microbiome diversity^59,60^. There have also been a few studies that have examined diversity differences between periodontal disease and healthy subjects in humans using 16s rRNA sequencing. But these studies suffer from a small sample size and the results are not clear in regard to bacterial diversity. For instance, Shi et al.^61^ reported an increase in diversity in subgingival bacterial communities from periodontitis patients, whereas Chen et al.^62^, Schwarzberg et al.^63^, and Acharya et al.^64^ showed no differences in the diversity of the individual subgingival or salivary microbiome and a lack of concordance in their changes after periodontal treatment. However, these studies are not directly comparable and have been conducted using 16s rRNA and not metagenome shotgun sequencing. Thus, other than the current study, there are no studies to date that have examined the mouse oral microbiome of periodontal disease using shotgun sequencing approaches. Shotgun sequencing methods examine the combined host and microbial DNA and do not enrich for bacterial DNA, whereas 16S sequencing methods specifically enrich for bacterial DNA. Thus, differences in ability to detect changes in bacterial diversity and total bacterial load may also stem from the use of different sequencing methods.

In summary, this study introduces a new polymicrobial mouse model of periodontal disease in a common mouse strain with an intact immune background. Further, this model makes use of four key periodontal pathogens that are introduced via oral inoculation; an improvement over mono-infection and ligature models. In addition, this model successfully recapitulates the bone loss and intrabony defects characteristic of periodontal disease. A unique and significant altered virome was associated with bone loss in this model of periodontal disease. Lastly, a notable finding afforded with this model and via the use of micro-CT analysis is that a polymicrobial oral infection can significantly impact periodontal ligament width/space at significant distances far removed from the site of infection and bone loss. This latter finding and the unique altered virome may have significant implications for the pathogenesis of periodontal disease and biomechanical properties of the periodontium.

## Methods

### Periodontal bacteria and polymicrobial inoculum

*P. gingivalis* FDC 381, *T. denticola* ATCC 35405, *T. forsythia* ATCC 43037, and *F. nucleatum* ATCC 10953 were obtained from ATCC (Manassus, VA) and cultured anaerobically at 37 °C^51^. *P. gingivalis and F. nucleatum* were cultured in Tryptic Soy Broth (Becton Dickinson, Franklin Lakes, NJ) containing 5 mg/ml yeast extract, 0.5 mg/ml l-cysteine hydrochloride, 5 μg/ml hemin, 1 μg/ml menadione and 5% fetal bovine serum (FBS) (Gibco Thermo Fisher Scientific, Waltham, MA) for 3 days. *T. denticola* was cultured in oral treponeme enrichment broth media (Anaerobe systems, Morgan Hill, CA) for 5 days. *T. forsythia* was grown in tryptic soy broth containing 5 mg/ml yeast extract, 0.5 mg/ml l-cysteine hydrochloride, 5 μg/ml hemin, 1 μg/ml menadione, 10 μg/ml *N*-acetylmuramic acid (Sigma-Aldrich, St. Louis, MO), and 5% FBS (Gibco) for 7 days. The concentration of each bacterium was determined quantitatively, and the organism was resuspended in phosphate-buffered saline (PBS) at 1 × 10^10^ bacteria per ml for experiments.

For the oral polymicrobial inoculation, *P. gingivalis* was gently mixed with an equal quantity of *T. denticola* and allowed to interact for 5 min. Subsequently, *T. forsythia* was added to the tubes containing *P. gingivalis* and *T. denticola*, and the bacteria were mixed gently for 1 min and allowed to interact for an additional 5 min. Lastly, *F. nucleatum* was added and mixed well with *P. gingivalis*, *T. denticola*, and *T. forsythia*. After 5 min, an equal volume of sterile 4% (w/v) carboxymethyl cellulose (CMC; Sigma-Aldrich) in PBS was added to the bacterial consortium and mixed thoroughly, then this mixture was used for the oral inoculation^51^.

### Infection of mice

A total of 12 eight-week-old BALB/cByJ female mice (The Jackson Laboratories, Bar Harbor, ME) were housed in microisolator plastic cages and randomly distributed into two groups (six mice per group); a control and an infection group (Fig. 1). The experimental protocols were approved by the Institutional Animal Care and Use Committee of the University of California, San Francisco (IACUC APPROVAL NUMBER: AN171564-01B). Briefly, all the mice were initially given trimethoprim (0.17 mg per ml) and sulfamethoxazole (0.87 mg per mL) daily for 7 days in the drinking water and then their oral cavity was rinsed with 0.12% chlorhexidine gluconate (Peridex, 3M, Maplewood, MN) mouth rinse to suppress the native oral microbiota^48^. The polymicrobial inoculum (5 × 10^9^ combined bacteria per ml; 1 × 10^9^ cells in 0.2 ml per mouse; 2.5 × 10^8^ *P. gingivalis*, 2.5 × 10 *T. denticola*, 2.5 × 10^8^ *T. forsythia*, and 2.5 × 10^8^ *F. nucleatum*) was administered topically in the morning for 4 consecutive days every week for a total of 8 weeks. A control solution without bacterial pathogens was administered as the control treatment.

Oral microbial swab samples were collected with micro cotton tips at different time points to evaluate microbial infection. Sample collection took place the day before antibiotic treatment, the day before infection and at 1, 4, and 8 weeks post infection. The post-infection samples were collected 3 days after each infection cycle (Fig. 1). The samples were collected by swabbing the oral cavity, teeth, and surrounding gingiva of the mice using a sterile micro sized cotton swab, which was then immersed in 10:1 Tris-EDTA buffer immediately and stored at −80 °C until further processing for DNA isolation.

After 8 weeks of polymicrobial infection, mice were euthanized and the blood was collected via cardiac puncture for analysis of antibody response to the periodontal pathogens. The maxillae and mandibles were resected from each mouse for various analyses.

### DNA isolation, ethanol precipitation, and real-time PCR to confirm bacterial infection

Oral swabs were used to evaluate and confirm infection in the mice. DNA was isolated and purified from the swabs using the QIAamp^®^ DNA Mini kit (Qiagen, Germantown, MD, USA) following manufacturers protocols. Specifically, the swabs were placed in a 2 ml microcentrifuge tube, then 600 μl of PBS was added to each sample tube. To this, 20 μl of QIAGEN proteinase K and 600 μl of Buffer AL were added to each sample, then samples were mixed immediately by vortexing for 15 s. Subsequently, samples were incubated at 56 °C for 10 min and briefly centrifuged. Then, 600 μl of ethanol (96–100%) was added to each sample, and samples were mixed by vortexing and briefly centrifuged. Subsequently, 700 μl of this mixture was added to the QIAamp Mini spin column and centrifuged at 6000 × *g* for 1 min. The QIAamp Mini spin column was placed in a clean 2 ml collection tube, and the tube containing the filtrate was discarded. Then the steps above were repeated by applying up to 700 μl of the remaining mixture to the QIAamp Mini spin column. Then 500 μl of Buffer AW1 were added and samples centrifuged at 6000 × *g* for 1 min. Next the QIAamp Mini spin columns were placed in a clean 2 ml collection tube and the collection tube containing the filtrate was discarded. Then 500 μl of Buffer AW2 was added and samples centrifuged at full speed (20,000 × *g*) for 3 min. The QIAamp Mini spin column was then placed in a new 2 ml collection tube and the old collection tube with the filtrate was discarded. This was centrifuged at full speed (20,000 × *g*) for 1 min. The QIAamp Mini spin column was placed in a clean 1.5 ml microcentrifuge tube and the collection tube containing the filtrate was discarded. Lastly, 150 μl of Buffer AE was added, sample incubated at room temperature for 1 min, then centrifuge at 6000 × *g* for 1 min.

Ethanol precipitation of DNA was then performed to prepare the sample for subsequent RT-PCR. Specifically, the following were added to each sample in sequential order; 1/10 volume of 3 M sodium acetate, pH 5.2. and 2.5 volumes of 100% Ethanol. Then 2 μL of a 15 mg/μL glycogen reagent (Glycoblue^TM^ Coprecipitant) was added to each sample as a coprecipitating agent. This was mixed by vortexing and samples frozen overnight at −20 °C. Then the sample underwent microcentrifugation at full speed (20,000 × *g*) at 4 °C for 1 h. The supernatant was carefully pipetted off, and 250 μL of 70% ethanol was added and the sample, and the sample centrifuged at full speed (20,000 × *g*) at 4 °C for 10 min. The pellets were dried at room temperature for 15 min, then 25 μL of Tris-EDTA buffer was added to the samples.

Standard RT-PCR was used to confirm the presence of the periodontal pathogens in the oral swabs. The four periodontal pathogens and total bacteria were quantified by RT-PCR using primers (Invitrogen; Table 4) corresponding to 16S ribosomal RNA. Tenfold serial dilutions of DNA of known concentration were used to construct standard curves for quantification of periodontal pathogens and total bacteria. The amplification was conducted using a QuantStudio 3 Real Time PCR system (Thermo Fisher Scientific) with a final reaction volume of 20 μL that included the PowerUp SYBR Green Master Mix (Thermo Fisher Scientific), DNA (15 ng/μL), and primers. The optimized thermal cycling conditions were as follows: 95 °C for 10 min followed by 50 cycles of denaturing at 95 °C for 15 s, annealing and extension at 60 °C for 1 min. The melt curve protocol followed with 15 s at 95 °C, and then 60 °C for 1 min and 95 °C for 1 s. Data were analyzed using QuantStudio^TM^ Design & Analysis Software v1.4.3 (Thermo).

**Table 4:** PCR primers used for confirmation of bacterial infection.

Bacterium	Primer sequence (5′-3′)
*Porphyromonas gingivalis*	F: AGT CGA GTT GCA GAC TCC GAT CC
	R: AAC CCA CAT CGG TAG TTG CTA ACA G
*Fusobacterium nucleatum*	F: AGA GTT TGA TCC TGG CTC AG
	R: GTC ATC GTG CAC ACA GAA TTG CTG
*Tannerella forsythia*	F: T CCC AAA GAC GCG GAT ATC A
	R: ACG GTC GCG ATG TCA TTG T
*Treponema denticola*	F: AGGGATATGGCAGCGTAGCA
	R: TTGCGGGACTTAACCCAACA
Universal (total bacteria)	F: TTGGGTTAAGTCCCGC
	R: ATCCCCACCTTCCTCC

### Serum antibody analysis

Blood was harvested from all mice the day of euthanasia then centrifuged at 200 × *g* for isolation of serum. Serum was then stored at −80 °C for subsequent assessment of a specific host response in the form of immunoglobulins (IgG) against *P. gingivalis*, *T. denticola*, *T. forsythia*, and *F. nucleatum* using an enzyme-linked immunosorbent assay (ELISA). For positive controls for the ELISA assay, each of these pathogens was grown to a cell density of 7 × 10^8^ cells/ml and harvested by centrifugation (7000 × *g*, 30 min, 4 °C). After washing, the cells were treated overnight with 0.5% formalin in buffered saline, then diluted to 0.3 (OD_600 nm_) in 0.05 M carbonate–bicarbonate buffer and used as the coating antigen. The amounts of specific IgG antibodies present were determined by using a mouse IgG ELISA quantification kit (Bethyl Laboratories, USA). A pilot test was first performed with serum diluted at 1:100 to confirm reactivity with the bacterial antigens. Then, after coating with the formalin-fixed bacterial cells, wells were incubated with diluted mouse serum (1:100) for 1 h at room temperature, washed with PBS containing 0.05% Tween-20 (PBST), and alkaline phosphatase-conjugated goat anti-mouse IgG and the 3,3′,5,5′-tetramethylbenzidine chromogenic substrate reagent were added for detection and measurement of antibody response. Samples were assayed in duplicate and the purified mouse IgG was used to establish a standard curve on each plate for specific IgG quantification.

### Immune-gene profiling PCR array

Twelve sacrificed mice had their right maxillary gingiva dissected and stored at −80 °C, six were control mice and six were infected or experimental mice. Tissue was powdered with a mortar and pestle under continuous liquid nitrogen. Total tissue RNA was isolated (RNeasy mini Kit; QIAGEN) and reversely transcribed (SuperScript VILO Master Mix; Thermo Fisher). Gene expression was assessed using the Mouse Common Cytokines RT^2^ Profiler PCR Array (QIAGEN) containing primers for 84 genes. Results were expressed as PCR cycles to threshold (Ct) and normalized to 1 control gene, glucuronidase beta (Gusb). Ct values were used for calculations of fold changes in gene expression according to the 2^−ΔΔCt^ method.

### Shotgun sequencing and microbiome/virome data production and analyses

The gingiva of 12 mice (6 controls and 6 infected) was collected and used for DNA extraction and isolation using the QIAamp^®^ DNA Mini kit (Qiagen, Germantown, MD, USA) according to manufacturer’s instructions. DNA purity and quantity was deemed suitable and met quality control measures for standard shotgun sequencing by Novogen, Inc. (en.novogene.com). Shotgun metagenome sequencing library preparation has been done by Novogen, Inc. and libraries have been sequenced on Illumina Hiseq4000 machines. FASTQ files were generated from the sequencing machines and used for the analyses of the microbiome and virome as described below.

### Processing raw data and data clean up

The following criteria were used for processing the raw data and cleaning. Low quality bases (*Q*-value ≤ 38), which exceeded a certain threshold (40 bp by default) were trimmed. Reads which contained N nucleotides over a certain threshold (10 bp by default) were trimmed. Reads which overlapped with adapter over a certain threshold (15 bp by default) were trimmed.

### Metagenome assembly

We utilized de novo assembly for each sample as follows. Samples passing quality control were assembled initially using SOAPdenovo (http://soap.genomics.org.cn/soapdenovo.html). The Scaffolds were cut off at “N” to get fragments without “N”, called Scaftigs. Clean data for all samples were mapped to assembled Scaftigs using SoapAligner (http://soap.genomics.org.cn/soapaligner.html) and unutilized paired-end reads were collected. Mixed assembly was conducted on the unutilized reads with the same assembly parameter. The scaftigs of each sample and mixed assembly, which were less than 500 bp, were trimmed.

### Taxonomy annotation

The following taxonomy annotation scheme was used. We aligned unigenes to the NCBI nonredundant database with DIAMOND to taxonomically annotate each metagenomic homolog (MEGAN). According to the abundance table of each taxonomic level, various analyses were performed using custom scripts by R and Python.

For the microbiome/virome analyses, two mice from the infection group were excluded from the analysis due to their lack of enough sequencing reads from microbial species. Species with average reads of less than 1 RPM (read per million) were filtered out. For the bacterial and viral content, the p-values were computed using a two-sample *t* test assuming equal variance of samples from the two groups. The Shannon diversity index was used to quantify the bacterial and viral diversity. We performed linear regression to quantify the association of bacterial and viral species, immune genes to bone loss and periodontal ligament space parameters, respectively. A log10 scale was used for bacterial and viral counts, while the original scale was used for other quantities. The Benjamini–Hochberg procedure was used for multiple testing correction and the corresponding values (FDR) are reported^65^.

### Morphometric analysis of alveolar bone loss

The left maxillae and mandible from each mice were carefully dissected, autoclaved, and mechanically de-fleshed to remove all the soft tissue. The maxillae and mandible were then immersed in 3% hydrogen peroxide overnight and stained with 1% methylene blue. For bone loss measurements, the distance between the cementoenamel junction (CEJ) and alveolar bone crest (ABC) were measured from a total of 28 sites on the buccal and lingual/palatal surfaces of the molars (3 sites on the first molar, 2 sites on the second molar, and another 2 sites on the third molar)^39,66^. Digital images of both buccal and lingual/palatal root surfaces of all molar teeth were captured under a stereo dissecting microscope (SMZ1000, Nikon) at the magnification shown in the images, then the line tool of Image J software (NIH Image) was used to measure the alveolar bone loss from the CEJ to ABC. Two blinded examiners (experienced periodontists) performed all measurements twice at separate times. Both horizontal bone loss and intrabony defects were detected under the stereo dissecting microscope. The intrabony defects were marked as present or absent.

### Micro-CT analysis of periodontal ligament space

Each specimen was scanned using X-ray micro-computed tomography (Micro-XCT 200, Carl Zeiss, Pleasanton, CA) at 4X magnification with LE#2 filter, 80 W of power, and 40 kV of voltage. X-ray projections were reconstructed into volumes utilizing XMReconstructor (Carl Zeiss, Pleasanton, CA). Reconstructed images were further processed by AVIZO^®^ software (2019.1, Thermo Fisher Scientific, Hillsboro, Oregon) to evaluate the PDL space between the roots of molars and respective alveolar bone sockets in control (without infection) and experimental (with infection) groups. The normalized frequency of PDL space for each specimen was plotted for control and experimental groups. The parameters for peak value and peak width were estimated through a Gaussian fit using MATLAB Curve Fitting Tools (R2019a, The MathWorks, Inc., Natick, MA). Student’s *t* test was used to obtain significant differences (*p* < 0.05) between groups with a 95% confidence interval.

### Statistical analysis

SPSS 21.0 statistical software (IBM, Chicago, IL, USA) was used for statistical analysis. Alveolar bone resorption and antibody analysis data were expressed as means ± standard deviations. Student’s *t* test was used to compare two independent groups. For the intrabony defects comparison, data were shown as frequency and percentage, and chi-square test was applied. Values of *p* < 0.05 were considered significant. The associations between bone loss, immune profile changes, microbiome/virome, and PDL width changes were quantified using linear regression. The Benjamini–Hochberg procedure was used for multiple testing correction and the corresponding FDR values were reported^65^.

### Reporting summary

Further information on research design is available in the Nature Research Reporting Summary linked to this article.

## Supplementary information

Supplemental Figures

Reporting Summary

## Data Availability

The data that support the findings of this study are available at the following site: https://figshare.com/projects/Polymicrobial_Periodontal_Disease_Triggers_a_Wide_Radius_of_Effect_and_Unique_Virome/74361.

## Code availability

The code for analyzing the microbiome data is provided along with the data at: https://figshare.com/articles/Data_Li_et_al_zip/11638008.
