# Supplementary material for: Polymicrobial periodontal disease triggers a wide radius of effect and unique virome
Source: NPJ Biofilms Microbiomes. 2020 Mar 10;6:10. doi: 10.1038/s41522-020-0120-7 (PMC7064479; doi:10.1038/s41522-020-0120-7)
Supplement: Supplementary file 1 — Supplemental Figures [file 41522_2020_120_MOESM1_ESM.pdf]

Supplemental Figure 1

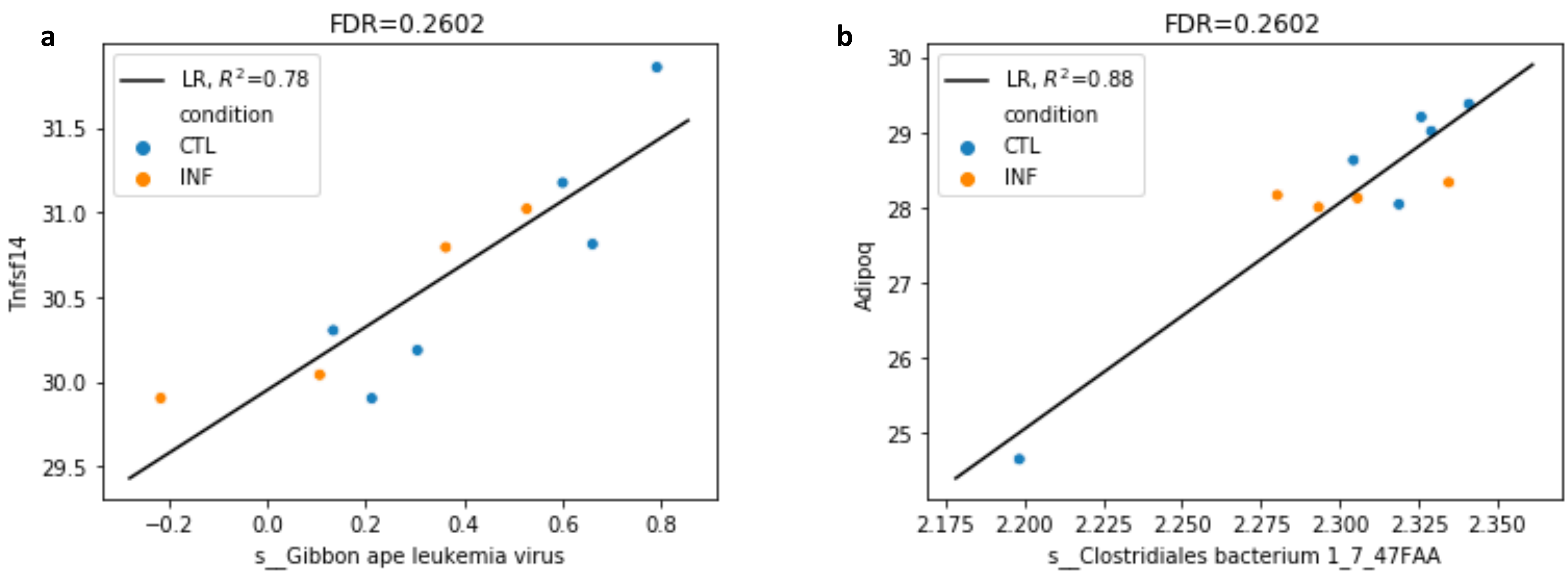

Supplemental Figure 1. Specific immune genes are associated with specific bacterial and viral species

The graphs show the top viral (a; Gibbon ape leukemia virus) and bacterial (b; Clostridiales Bacterium) species that are associated with immune genes in this model. The X-axes show the abundance of microbes/viruses and the Y-axes show the level gene expression of the immune genes.

Supplemental Figure 2

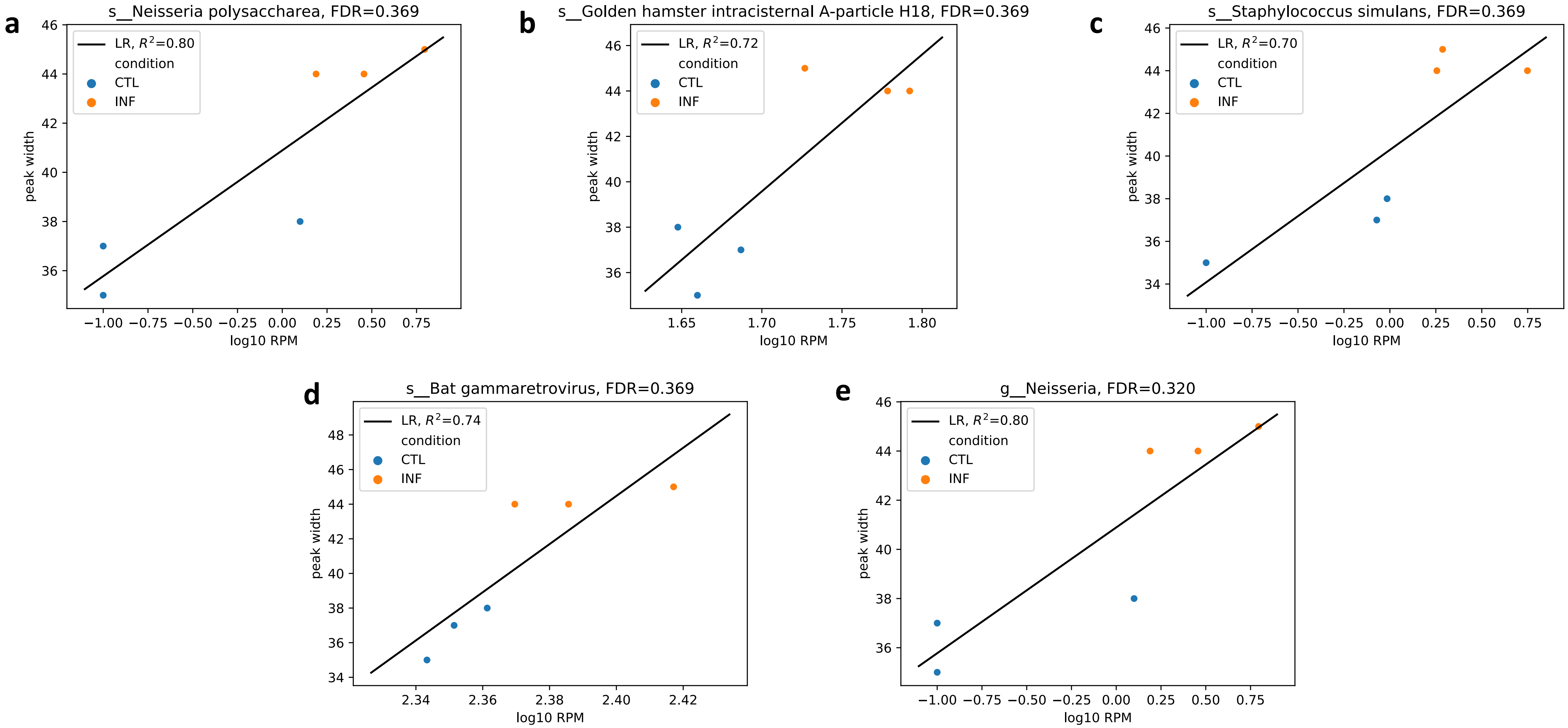

Supplemental Figure 2. Specific viruses and bacteria are associated with increased PDL space

The graphs show the association between several viruses and bacteria (genus and species) and PDL space (a-e) . The X-axes show the peak PDL width and the Y-axes show the viruses and bacterial species and genus.
